# Noninvasive optical characterization of muscle blood flow, oxygenation, and metabolism in women with fibromyalgia

**DOI:** 10.1186/ar4079

**Published:** 2012-11-01

**Authors:** Yu Shang, Katelyn Gurley, Brock Symons, Douglas Long, Ratchakrit Srikuea, Leslie J Crofford, Charlotte A Peterson, Guoqiang Yu

**Affiliations:** 1 Center for Biomedical Engineering, University of Kentucky, Lexington, KY 40506, USA; 2 Department of Gerontology, College of Public Health, University of Kentucky, Lexington, KY 40536, USA; 3 College of Health Sciences, University of Kentucky, Lexington, KY 40536, USA; 4 Department of Physiology, Faculty of Science, Mahidol University, Bangkok 10400, Thailand; 5 Department of Internal Medicine, College of Medicine, University of Kentucky, Lexington, KY 40536, USA

## Abstract

**Introduction:**

Women with fibromyalgia (FM) have symptoms of increased muscular fatigue and reduced exercise tolerance, which may be associated with alterations in muscle microcirculation and oxygen metabolism. This study used near-infrared diffuse optical spectroscopies to noninvasively evaluate muscle blood flow, blood oxygenation and oxygen metabolism during leg fatiguing exercise and during arm arterial cuff occlusion in post-menopausal women with and without FM.

**Methods:**

Fourteen women with FM and twenty-three well-matched healthy controls participated in this study. For the fatiguing exercise protocol, the subject was instructed to perform 6 sets of 12 isometric contractions of knee extensor muscles with intensity steadily increasing from 20 to 70% maximal voluntary isometric contraction (MVIC). For the cuff occlusion protocol, forearm arterial blood flow was occluded via a tourniquet on the upper arm for 3 minutes. Leg or arm muscle hemodynamics, including relative blood flow (rBF), oxy- and deoxy-hemoglobin concentration ([HbO_2_] and [Hb]), total hemoglobin concentration (THC) and blood oxygen saturation (StO_2_), were continuously monitored throughout protocols using a custom-built hybrid diffuse optical instrument that combined a commercial near-infrared oximeter for tissue oxygenation measurements and a custom-designed diffuse correlation spectroscopy (DCS) flowmeter for tissue blood flow measurements. Relative oxygen extraction fraction (rOEF) and oxygen consumption rate (rVO_2_) were calculated from the measured blood flow and oxygenation data. Post-manipulation (fatiguing exercise or cuff occlusion) recovery in muscle hemodynamics was characterized by the recovery half-time, a time interval from the end of manipulation to the time that tissue hemodynamics reached a half-maximal value.

**Results:**

Subjects with FM had similar hemodynamic and metabolic response/recovery patterns as healthy controls during exercise and during arterial occlusion. However, tissue rOEF during exercise in subjects with FM was significantly lower than in healthy controls, and the half-times of oxygenation recovery (Δ[HbO_2_] and Δ[Hb]) were significantly longer following fatiguing exercise and cuff occlusion.

**Conclusions:**

Our results suggest an alteration of muscle oxygen utilization in the FM population. This study demonstrates the potential of using combined diffuse optical spectroscopies (i.e., NIRS/DCS) to comprehensively evaluate tissue oxygen and flow kinetics in skeletal muscle.

## Introduction

Fibromyalgia (FM) is a common chronic widespread pain syndrome affecting approximately 2 to 5% of the US population [1,2]. Historically, older women are more susceptible to FM compared to men or young women [2-4]. People suffering from FM have symptoms of increased muscle fatigue and reduced tolerance to exercise, similar to patients with chronic fatigue syndrome (CFS) [5]. Although the specific pathogenic mechanisms of FM remain unclear, studies have suggested that the muscle pain and fatigue of FM may be associated with mitochondrial dysfunction [6], lower capillary density [7,8], reduced capillary permeability [9], or impaired vasodilatory capacity [10,11]. Those impairments may consequently affect muscle tissue microcirculation and oxygen metabolism. However, previous studies investigating peripheral/muscle blood flow or oxygen consumption in populations with FM have reported conflicting results [8,11-17]. Some studies have found reduced skin/muscle blood flow or oxygen consumption in people with FM [8,11,12,14,15,17], whereas others reported that muscle blood flow or oxygen metabolism was not significantly altered by FM [13,16]. It has also been reported that subjects with FM have prolonged oxygen level (oxy- and deoxyhemoglobin concentrations) recovery times following muscle ischemia [18] or aerobic exercise [19]. The previous studies provide incomplete information, and simultaneous measurements of tissue blood flow, blood oxygenation and oxygen metabolism are required for a more comprehensive evaluation of dynamic skeletal muscle and circulatory functions in FM.

Methods previously used to measure muscle blood flow, oxygenation and oxygen consumption in FM population all have limitations. Laser Doppler cannot detect blood flow in deep muscle tissue, and is limited to superficial layers (such as skin) [8]. The Xe^133 ^technique [12,16,20] or contrast media-enhanced color ultrasound Doppler [17] can measure microvasculature blood flow in deep muscle tissue; however the invasive and complex procedure of injecting radioactive isotopes or contrast agents limits their widespread use in the clinic. Partial pressure of oxygen (PO_2_) electrodes have been used to invasively assess muscle oxygenation in a tiny spot [21], which may not be representative of the whole skeletal muscle. Phosphorus magnetic resonance spectroscopy (P-31 MRS) has been used to assess muscle oxygen consumption [14], but does not provide high temporal resolution and requires large and expensive instrumentation [22].

Near-infrared diffuse optical spectroscopy (NIRS) offers a noninvasive, rapid, portable, and low-cost alternative for monitoring tissue blood oxygenation and oxygen consumption in microvasculature, although it does not directly measure tissue blood flow. Near-infrared light probes tissue millimeters to centimeters below the skin surface, allowing for measurement of oxy- and deoxyhemoglobin concentrations ([HbO_2_] and [Hb]), total hemoglobin concentration (THC) and blood oxygen saturation (StO_2_) [23]. NIRS has been broadly used for noninvasive assessment of tissue oxygenation in clinic. According to a review paper written by Ferrari *et al. *[24], approximately160 articles on the use of NIRS to study muscle physiology (primarily in upper and lower limb muscles) were published from 2007 up to the end of 2010 [24]. NIRS has also been used in studies of FM [18,19] and CFS [5,25] to evaluate tissue hemodynamic responses following muscle ischemia and aerobic exercise. Near-infrared diffuse correlation spectroscopy (DCS) is an emerging technique capable of directly and noninvasively measuring microvascular blood flow in various tissues, including human skeletal muscles [23,26-28]. DCS combines several attractive features for blood flow measurement including noninvasiveness, high temporal resolution (up to several milliseconds) [29], portability, and relatively large penetration depth (up to several centimeters) [30,31]. DCS for blood flow measurement in various organs and tissues have been validated to other standards, including laser Doppler [32,33], Xenon-CT [34], fluorescent microsphere flow measurement [35], and perfusion magnetic resonance imaging (perfusion MRI) [36]. Recently, our group has developed a hybrid diffuse optical instrument which combines a commercial NIRS tissue oximeter (Imagent, ISS Inc., IL, USA) and a custom-designed DCS tissue flowmeter for measurements of both tissue blood flow and blood oxygenation [23]. Simultaneous measurements of blood flow and oxygenation enable estimation of the oxygen metabolic rate in tissue [27], thus providing comprehensive information about dynamic tissue oxygen kinetics.

The present study aims to use the hybrid NIRS/DCS instrument to evaluate skeletal muscle tissue hemodynamics (blood flow and oxygenation) and oxygen metabolism in postmenopausal women with and without FM. Because the abnormality of oxygen kinetics in FM may not be apparent at rest, leg skeletal muscle hemodynamics and metabolism were manipulated by isometric fatiguing exercise. In addition, a protocol of temporary cuff occlusion was used to create muscle ischemia in the forearm and to monitor muscle blood flow and oxygen recovery dynamics during reactive hyperemia. To the best of our knowledge, this study quantified for the first time, skeletal muscle hemodynamics and metabolism simultaneously in subjects with FM and well-matched healthy controls during exercise and during a muscle ischemic challenge. We hypothesize that FM affects muscle hemodynamic/metabolic responses to fatiguing exercise and ischemic challenge, which can be detected by our hybrid NIRS/DCS instrument. This study provided comprehensive and comparative evaluation of muscle oxygen kinetics to improve the understanding of the physiological mechanisms of FM.

## Materials and methods

### Study protocols

The study was reviewed and approved by the University of Kentucky Institutional Review Board. Thirty-seven women between the ages of 51 and 70 years participated in this study, including 14 women with FM and 23 healthy controls. All subjects gave signed consent prior to the study. FM was diagnosed in accordance with the 1990 criteria established by the American College of Rheumatology [37]. Subjects with FM and healthy controls were matched in age, height, weight, body mass index (BMI), physical activity and baseline maximal voluntary isometric contractions (MVIC). Physical activity was characterized by the international physical activity questionnaire (IPAQ) [38] while baseline MVIC was determined prior to fatigue exercise (described later in this section). The duration of FM (that is, the time interval between the diagnosis of FM and the start of the fatiguing exercise study) was 13.69 ± 1.90 years (mean ± standard error, SE), ranging from 3 to 24 years. Some subjects with FM were taking medications such as anti-inflammatory drugs, low-dose aspirin, and fish oil supplements. These medications were discontinued at least 3 days prior to exercise to minimize the impact on muscle hemodynamic responses.

Two experimental protocols were used to investigate muscle hemodynamic/metabolic responses, including fatiguing leg isometric contractions and arm cuff occlusion (muscle ischemic challenge). Each subject was asked to perform fatiguing leg exercise followed by cuff occlusion in the arm to minimize the potential interference between the two protocols in a single muscle, and to examine responses to both exercise and ischemia. Leg knee extensor muscles were evaluated for fatiguing exercise since these muscles are primarily used for daily activities, and the subjects were tolerant to the protocol. In addition, previous studies in FM [18,19] have utilized a cuff occlusion protocol using the forearm muscles (flexor carpi radialis) to investigate oxygen kinetics, allowing a basis for comparison.

At least 3 days prior to fatiguing exercise, each subject participated in a session to become familiar with the performance of MVIC on a Biodex multi-joint dynamometer (System 4, Biodex Medical Systems Inc., NY, USA) as follows: the subject was seated in an upright position with the seat tilted at an angle of 85°. The lateral femoral epicondyle was aligned to the center of the dynamometer shaft. Stabilization was provided by two shoulder straps, and a waist strap was used to minimize the use of skeletal muscles other than the knee extensor. Each subject's testing foot was secured by a strap to the dynamometer with a fixed knee angle of 90°. The subject was then instructed to perform leg isometric contractions (that is, kicking against the dynamometer lever arm) held for 3 to 4 seconds, while the maximal value of the torque generated during the isometric contraction was recorded. During MVIC, an electrical stimulation (ES) was used to noninvasively induce superimposed force to the muscle via surface electrodes over the proximal and distal portions of the thigh [39]. When applying ES to the muscle, all motor units are recruited. Any increment in force from ES would suggest incomplete activation of the muscle from MVIC. After performing MVIC measures, the central activation ratio (CAR) was quantified using the following equation:

CAR = MVIC/total force, where the total force = MVIC + superimposed twitch [40].

The isometric fatiguing exercise was performed on the dynamometer and started with three MVIC trials with a 3-minute rest between MVICs. Following the MVIC tests, an optical sensor was secured by medical tape over the vastus lateralis (mid belly) at the mid thigh of the evaluated leg, and a 3-minute baseline measurement was recorded. Following the baseline measurement, the subject was instructed to perform six sets of twelve isometric muscle contractions at a 40% duty cycle (4-second contraction, 6-second rest) steadily increasing from an initial intensity of 20% MVIC and eventually reaching an intensity of 70% of the MVIC with a increment of 10% MVIC after each set. While performing exercise, the subject received visual feedback by looking at the targeted intensity level shown on a computer and was encouraged to achieve each set intensity (20 to 70% MVIC). Between sets of isometric muscle contractions, a single MVIC was performed as the primary measure of fatigue. There was no additional rest between sets. In total, 78 muscle contractions (12 × 6 contractions + 6 MVIC) were performed during the course of fatiguing exercise. Fatigue and pain during exercise were evaluated according to the visual analog scale (VAS); the subject was asked to indicate her pain/fatigue severity on a 100-mm scale. Details about the fatigue and pain questionnaires can be found in references [41] and [42]. A higher VAS score indicates more pain or fatigue. After completion of the exercise, the subject was asked to perform one MVIC to evaluate strength recovery at time points 3, 6, 9 and 12 minutes.

The cuff occlusion protocol in the forearm started approximately 10 minutes after the fatiguing leg exercise. The participant sat in an upright position and the right arm was extended resting on a horizontal support. A fast-inflating automatic tourniquet cuff (ATS 1000, Zimmer Inc., IN, USA) was placed on the upper arm and an optical sensor was secured by medical tape over the flexor carpi radialis muscle. After a 3-minute baseline measurement, arterial blood flow was occluded via the tourniquet on the upper arm at a pressure of 230 mmHg for 3 minutes. The pressure was then released and measurements continued for an additional 5-minute recovery period.

### Hemodynamic/metabolic measurements

The optical sensor was connected to a hybrid diffuse optical instrument, which combined a commercial NIRS oximeter (Imagent, ISS Inc., IL, USA) for tissue oxygenation measurement and a custom-designed diffuse correlation spectroscopy (DCS) flowmeter for tissue blood flow measurement. The principle of the hybrid instrument has been described elsewhere [23,43]. Briefly, near-infrared laser light was delivered into the thigh or arm muscle, and the reflectance light was received by the photon detectors through source and detector fibers placed on the skin surface. The NIRS oximeter measured the amplitudes and phases of frequency-modulated light (110 MHz) at two wavelengths (830 and 690 nm) and four source-detector separations (2.0, 2.5, 3.0 and 3.5 cm) to extract tissue absorption and scattering coefficients [23,44]. Absolute values of [HbO_2_] and [Hb] were extracted from the measured tissue absorption coefficients at the two wavelengths [44]. Total hemoglobin concentration (THC) was then calculated as the sum of [HbO_2_] and [Hb], while tissue blood oxygen saturation (StO_2_) was calculated as 100% × [HbO_2_]/THC.

Preliminary data analyses indicated that the measured time courses of absolute tissue blood oxygenation, determined by the light amplitudes and phases from all four source-detector separations were too noisy to determine reliable time intervals for characterizing oxygen recovery, due to the unstable phase slopes over time. Thus, to evaluate tissue blood oxygenation recovery, we used the measured light amplitudes at the two wavelengths from a single source-detector separation (2.5 cm for arm muscles or 3.0 cm for leg muscles) to calculate the changes of [HbO_2_] and [Hb] (that is, Δ[HbO_2_] and Δ[Hb]) relative to their baselines (before physiological manipulations), based on the modified Beer-Lambert Law [45].

Blood flow index was extracted by fitting the autocorrelation curve determined from the detected temporal fluctuation of light intensity measured by DCS [23,27,30]. The unit of the blood flow index is cm^2^/s. Although this unit is different from the classical blood flow unit in biological tissues (ml/min/100 ml), its percentage changes have been found to correlate well with the blood flow changes measured by many other established modalities [32-36]. The relative blood flow (rBF) was then calculated by normalizing/dividing the blood flow index to its baseline. As with the NIRS oximeter, the source-detector separation used for DCS measurement was 2.5 or 3.0 cm for arm or leg muscle, respectively. The distal tips of source and detector fibers for NIRS oximeter and DCS were embedded in a foam pad to form a hybrid optical sensor [23,43]. The NIRS oximeter and DCS flowmeter were operated alternately via triggers controlled by a computer. For both protocols of fatiguing exercise and cuff occlusion, muscle blood flow and blood oxygenation were continuously monitored by the hybrid optical instrument with a frame sampling time of approximately 3 seconds throughout the experiments.

Relative (normalized to baseline) oxygen extraction fraction (rOEF) and oxygen consumption rate (rVO_2_) during fatiguing exercise were calculated based on Fick's law using the measured blood flow and oxygenation data respectively [27]:

rOEF = 100% × (1-StO_2_)/(1-StO_2baseline_) and rVO_2 _= 100% × rBF × rOEF, where StO_2baseline _represents the absolute baseline value of tissue blood oxygen saturation before exercise. Similar to the rOEF and rVO_2_, relative oxygenation changes were calculated by normalizing their absolute values to baselines respectively, resulting in r[HbO_2_], r[Hb], rTHC and rStO_2_. Throughout this paper, 'r' represents the relative value normalized/divided by its baseline, and 'Δ' (for example, Δ[HbO_2_] and Δ[Hb]) represents the subtracted difference between the time course data and its baseline.

### Data analysis

Since the optical signals during exercise were easily contaminated by the muscle fiber motion during leg contractions [26], hemodynamic/metabolic responses during fatiguing exercise were estimated by averaging the optical data over the 6 seconds immediately after fatiguing exercise. This very short post-exercise measurement period was selected as the most accurate reflection of the exercise state since rapid hemodynamic changes occur in muscle immediately after exercise [46,47]. Approximately one minute after exercise, most hemodynamic data became stable, allowing us to average a longer period of data acquisition to obtain a better signal-to-noise ratio. Therefore, hemodynamic data at the time points 3, 6, 9 and 12 minutes after exercise were quantified by averaging 30 seconds of data immediately preceding each time point. These data were then normalized to their pre-exercise baselines to evaluate responses during and post-exercise.

Hemodynamic recovery (that is, rBF, Δ[HbO_2_], Δ[Hb]) after fatiguing exercise or cuff occlusion was characterized by the recovery half-time [18,19,48], which was defined as the time interval from the end of occlusion/exercise to the time by which tissue hemodynamics had recovered to the half-maximal value. We used Δ[HbO_2_] and Δ[Hb] from one single source-detector separation (2.5 cm for arm muscles or 3.0 cm for leg muscles) to determine the half-times of oxygenation recovery. Note that the rOEF and rVO_2 _data had similar noise levels as the absolute blood oxygenation data because they were calculated from the absolute StO_2_. Thus, the recovery half-times for THC, StO_2_, rOEF and rVO_2 _are not reported in this study.

Average hemodynamic/metabolic responses by group are presented as means ± SE (error bars) in figures. The Student's *t*-test was used to compare differences in hemodynamic/metabolic data between the FM and healthy control groups. Linear regression was used to investigate the correlations among demographic, physical activity, strength and optical data.

## Results

During the familiarization MVIC test, the CAR was found to be very high and was not different between the subjects with FM and healthy controls (0.99 ± 0.01 vs. 0.99 ± 0.02, *P *= 0.90), indicating that both groups exerted full effort to perform the MVICs. There were no differences between the two groups in age, height, weight, BMI, IPAQ, or baseline MVIC (Table 1). Overall, the subjects lost an average of 28.9% strength after exercise, and there was no significant difference (*P *> 0.05) between groups in the percentage of strength loss. However, subjects with FM reported significantly more fatigue and pain during exercise (*P *< 0.001) compared to healthy controls (Table 1), according to the VAS.

**Table 1:** Subject characteristics

Parameters	Women with fibromyalgia (FM)(*n *= 14)	Healthy controls(HC) (*n *= 23)	*P*-value
Age (years)	60.0 ± 1.8	56.8 ± 1.0	0.09
Height (cm)	162.8 ± 1.5	163.4 ± 1.3	0.75
Weight (kg)	68.5 ± 4.3	69.1 ± 2.0	0.88
Body mass index (kg m^-2^)	25.7 ± 1.3	25.9 ± 0.8	0.87
FM duration (year)	13.7 ± 1.9	N/A	N/A
IPAQ (MET minute week^-1^)	7574 ± 1882	5928 ± 864	0.37
Baseline MVIC (N m)	113.6 ± 5.3	128.4 ± 5.6	0.08
% Loss in MVIC immediately post-exercise	31.4 ± 1.3	27.4 ± 2.4	0.23
% Loss in MVIC at 12 minutes post-exercise	21.1 ± 2.5	17.5 ± 2.0	0.28
VAS pain score pre-exercise (mm)	52.4 ± 6.5	6.9 ± 2.1	< 0.001
VAS pain score post-exercise (mm)	56.2 ± 7.0	17.4 ± 4.5	< 0.001
VAS pain score after 12 minutes recovery (mm)	53.6 ± 6.7	10.1 ± 3.3	< 0.001
VAS fatigue score post-exercise (mm)	61.0 ± 7.0	22.8 ± 4.2	< 0.001

Results are presented as mean ± standard error. 1 MET (metabolic equivalent of task), 1 kilocalorie kg^-1 ^hour^-1^; IPAQ, International Physical Activity Questionnaire; MVIC, maximal voluntary isometric contraction; VAS, visual analog scale.

Among the thirty-seven subjects (twenty-three healthy controls and fourteen subjects with FM), five subjects (three healthy controls and two subjects with FM) did not have oxygenation measurements due to NIRS oximeter instrument failure. Thus, all subjects were included for relative blood flow (rBF) analysis, whereas only 32 subjects (20 healthy controls and 12 subjects with FM) were included for analysis of blood oxygenation/oxygen metabolism. None of the baseline hemodynamic/metabolic variables were correlated with subject demographic data (age, height, weight, BMI), IPAQ, or baseline MVIC.

### Hemodynamic/metabolic changes following leg fatiguing exercise

**Figure 1:**
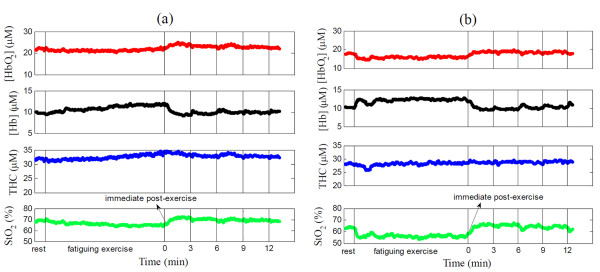
**Illustrative thigh muscle oxygenation responses throughout fatiguing exercise in (a) a subject with fibromyalgia (FM) and (b) a healthy control**. The oxygenation responses include oxy- and deoxyhemoglobin concentration ([HbO_2_] and [Hb]), total hemoglobin concentration (THC) and oxygen saturation (StO_2_), all presented in absolute values. The first two vertical lines indicate the beginning and the end of fatiguing exercise respectively, and the last four vertical lines indicate the time points 3, 6, 9 and 12 minutes after exercise. The arrow indicates the time points immediately post-exercise (over 6 seconds). Note that the muscle motion artifacts during exercise and during maximal voluntary isometric contraction (MVIC) tests at time points 3, 6, 9 and 12 minute post-exercise may contaminate optical measurements, as seen from the peaks in the figure.

**Figure 2:**
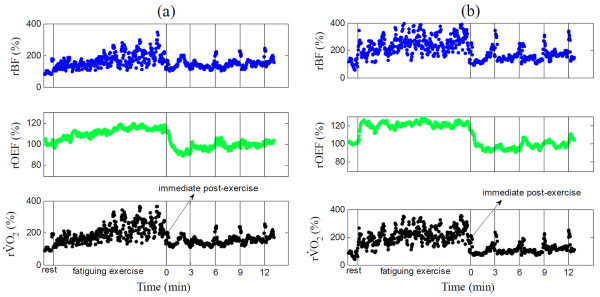
**Illustrative relative blood flow (rBF), oxygen extraction fraction (rOEF) and oxygen consumption rate (rVO_2_) throughout fatiguing exercise in (a) a subject with firbromyalgia (FM) and (b) a healthy control, all presented in percentage relative to baseline (%)**. The first two vertical lines indicate the beginning and the end of fatiguing exercise respectively, and the last four vertical lines indicate the time points 3, 6, 9 and 12 minutes after exercise. The arrow indicates the time points immediately post-exercise (over 6 seconds). Note that the muscle motion artifacts during exercise and during maximal voluntary isometric contraction (MVIC) tests at time points 3, 6, 9 and 12 minute post-exercise may contaminate optical measurements, as seen from the peaks in the figure.

Figure 1 ([HbO_2_], [Hb], THC, StO_2_) and Figure 2 (rBF, rOEF, rVO_2_) illustrate hemodynamic and metabolic responses in the knee extensor muscle of a subject with FM (Figure 1a, Figure 2a) and a healthy control (Figure 1b, Figure 2b) throughout fatiguing exercise. Subjects with FM and healthy controls had similar hemodynamic and metabolic response patterns. During exercise, rBF increased to meet the increase in oxygen demand (rVO_2_). The increased blood flow brought a greater volume of blood to the exercising muscles, thus elevating THC. Oxygen consumption in an exercising muscle resulted in an increase in [Hb] and decreases in [HbO_2_] and StO_2_. Once the exercise was stopped, all variables recovered towards their baseline values, which was mainly due to the rapidly decreased oxygen demand post-exercise. Notice that because muscle fiber motion artifacts during exercise affected optical measures, data were averaged over 6 seconds immediately following exercise (see the arrows in Figure 1 and Figure 2) to represent hemodynamic/metabolic responses during exercise.

**Figure 3:**
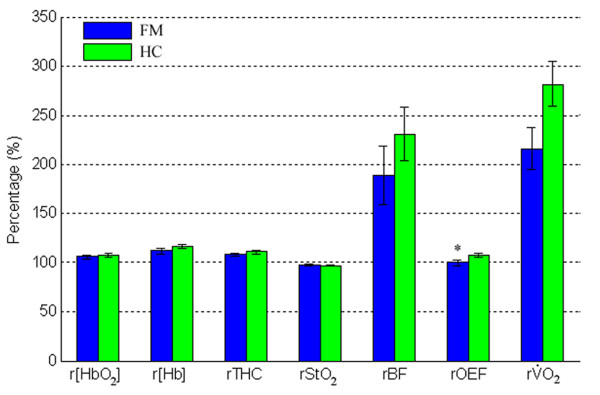
**Six-second average data immediately after fatiguing exercise (see the arrows in Figure 1 and Figure 2) as a measure of exercise-induced hemodynamic responses**. All parameters were normalized/divided to their baselines (%), resulting in relative change of oxy- and deoxyhemoglobin concentration(r[HbO_2_] and r[Hb]), total hemoglobin concentration (rTHC), oxygen saturation (rStO_2_), blood flow (rBF), oxygen extraction fraction (rOEF) and oxygen consumption rate (rVO_2_). The Student's *t*-test was used to compare the average rBF (n_HC _= 23, n_FM _= 14), r[HbO_2_], r[Hb], rTHC, rStO_2_, rOEF and rVO_2 _(n_HC _= 20, n_FM _= 12) in subjects with fibromyalgia (FM) and healthy controls (HC) immediately after fatiguing exercise. *P < 0.05.

The averaged percentage changes during exercise are shown by group in Figure 3. On average, subjects with FM tended to have smaller changes (assigned baseline to be 100%) in all measured variables during exercise than healthy controls, although most of the differences between the two groups were not significant. The increases in rBF and rVO_2 _during exercise were much larger than those in r[HbO_2_], r[Hb], rTHC, rStO_2 _and rOEF, leading to relatively larger variations (error bars) in rBF and rVO_2. _The rOEF during exercise was significantly less in subjects with FM compared to healthy controls (99.7 ± 2.6 vs. 107.4 ± 2.0; *P *= 0.03). No significant differences in any hemodynamic/metabolic variables were found between the two groups at time points 3, 6, 9 and 12 minutes after exercise (data not shown).

### Recovery half-time following leg fatiguing exercise

**Figure 4:**
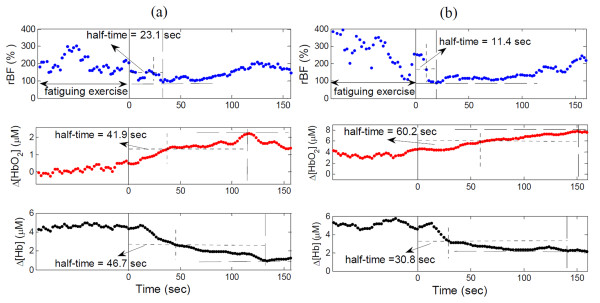
**Illustrative recovery half-times of relative blood flow (rBF), change in oxyhemoglobin concentration (Δ[HbO_2_]) and change in deoxyhemoglobin concentration (Δ[Hb]) following fatiguing exercise in (a) a subject with fibromyalgia (FM) and (b) a healthy control**. The vertical solid lines indicate the ending of exercise. The horizontal dashed and dotted lines indicate the maximal and half-maximal recovery values of hemodynamic variables, respectively. The vertical dotted lines indicate the recovery half-times.

**Figure 5:**
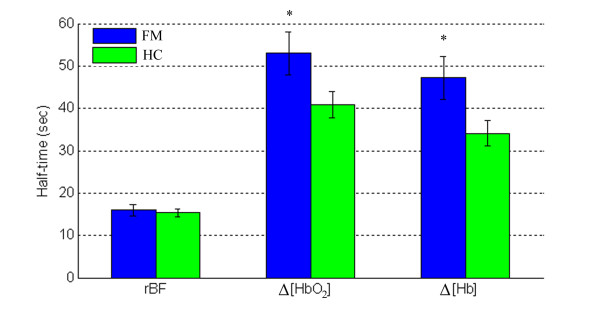
**Average recovery half-times of relative blood flow (rBF) (n_HC _= 23, n_FM _= 14), change in oxyhemoglobin concentration (Δ[HbO_2_]) and change in deoxyhemoglobin concentration (Δ[Hb]) (n_HC _= 20, n_FM _= 12) in subjects with fibromyalgia (FM) and healthy controls (HC) following fatiguing exercise**. The Student's *t*-test was used to compare the half-times between FM and healthy subjects. *P < 0.05.

Figure 4 illustrates hemodynamic recovery response of a subject with FM (Figure 4a) and a healthy control (Figure 4b) following the fatiguing exercise. Although large individual variation existed, subjects with FM and healthy controls showed similar hemodynamic recovery patterns; rBF and Δ[Hb] decreased, whereas Δ[HbO_2_] increased following the fatiguing exercise. The recovery half-time of rBF was shorter than oxygenation recovery for both the FM and control groups. On average (Figure 5), subjects with FM demonstrated a longer recovery half-time (s) than healthy controls in Δ[HbO_2_] (53.0 ± 5.1 vs. 40.7 ± 3.0; *P *= 0.03) and Δ[Hb] (47.1 ± 3.4 vs. 34.1 ± 2.8; *P *= 0.007), but not in rBF (15.9 ± 1.2 vs. 15.3 ± 0.8; *P *= 0.69). Notice that due to large inter-subject variations, the differences observed in some of the recovery times (for example, Δ[HbO_2_]) between individuals (Figure 4) may not agree with those between groups (Figure 5).

### Recovery half-time following arm cuff occlusion

**Figure 6:**
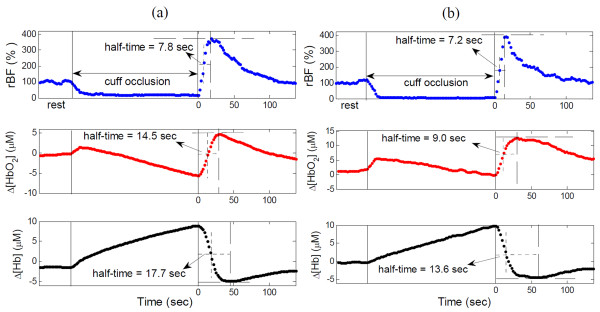
**Illustrative recovery half-times of relative blood flow (rBF), change in oxyhemoglobin concentration (Δ[HbO_2_]) and change in deoxyhemoglobin concentration (Δ[Hb]) following arm cuff occlusion in (a) a subject with fibromyalgia (FM) and (b) a healthy control**. The two solid vertical lines indicate the beginning and ending of cuff occlusion. The horizontal dashed and dotted lines indicate the maximal and half-maximal recovery values of hemodynamic variables, respectively. The vertical dotted lines indicate the recovery half-times.

**Figure 7:**
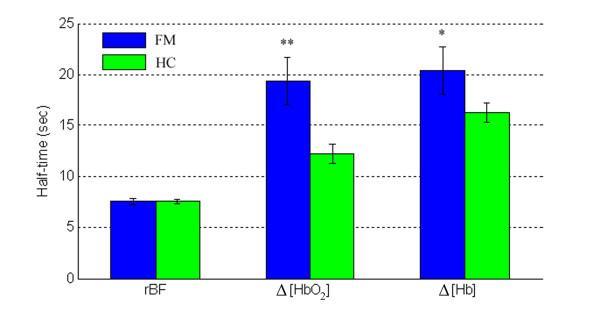
**Average recovery half-times of relative blood flow (rBF) (n_HC _= 23, n_FM _= 14), change in oxyhemoglobin concentration (Δ[HbO_2_]) and change in deoxyhemoglobin concentration (Δ[Hb]) (n_HC _= 20, n_FM _= 12) in subjects with fibromyalgia (FM) and healthy controls (HC) following arm cuff occlusion**. The Student's *t*-test was used to compare the half-times between FM and healthy subjects. *P < 0.05; **P < 0.005.

Figure 6 illustrates hemodynamic recovery responses of a subject with FM (Figure 6a) and a healthy control (Figure 6b) following arm cuff occlusion. Similar to the fatiguing exercise, healthy controls showed a similar hemodynamic recovery pattern to FM subjects. Following the release of the cuff, rBF and Δ[HbO_2_] increased, whereas Δ[Hb] decreased. The recovery half-time of rBF was shorter than oxygenation recovery for both groups. However, the recovery times of Δ[HbO_2_] and Δ[Hb] following cuff occlusion differed significantly between the two groups (Figure 7); FM demonstrated a longer recovery half-time (s) than healthy controls in Δ[HbO_2_] (19.4 ± 2.3 vs. 12.2 ± 0.9; *P *= 0.002) and Δ[Hb] (20.4 ± 1.8 vs. 16.3 ± 1.1; *P *= 0.04), but not in rBF (7.5 ± 0.3 vs. 7.6 ± 0.2; *P *= 0.86). These results mirror the data for fatiguing exercise.

## Discussion

Although previous studies have individually measured skeletal muscle blood flow [8,12,16,17,20], oxygenation [18,19,21] or oxygen consumption [14] in the FM population, none have investigated all these variables simultaneously in a single study. While NIRS and DCS have provided a great deal of hemodynamic data individually, both must be used in order to evaluate rOEF and rVO_2_. The present study reports the first results using the novel hybrid diffuse optical instrument for simultaneous monitoring of muscle blood flow, oxygenation during fatiguing leg exercise and during arm cuff occlusion, from which muscle oxygen extraction and consumption rate were derived. Both protocols, cuff occlusion and fatiguing exercise, created an imbalance between tissue oxygen supply (blood flow) and oxygen consumption for challenging muscle function. Cuff occlusion is a static protocol which creates tissue ischemia during occlusion and reactive hyperemia after release of occlusion. Fatiguing exercise is a dynamic exercise protocol which tests muscle regulatory and metabolic responses to stimulus (that is, exercise). Both protocols have been widely used to assess a variety of diseases affecting skeletal muscle, including peripheral arterial disease [27], CFS [5], and FM [18,19]. We adopted both protocols in the present study to extensively evaluate muscle oxygen and flow kinetics in both healthy and FM populations. We demonstrated in this study that the hybrid instrument has high sensitivity in detecting hemodynamic and metabolic responses to muscle ischemia/reperfusion and fatiguing exercise.

Subjects with FM had similar hemodynamic and metabolic response/recovery patterns to healthy controls during exercise and during arterial occlusion, and most measured variables did not show significant differences between the two groups. However, we observed that rOEF during exercise in subjects with FM were significantly lower than those in healthy controls (see Figure 3), and the half-times of oxygenation recovery (Δ[HbO_2_] and Δ[Hb]) were significantly longer (see Figure 5 and Figure 7).

Both lower rOEF and longer oxygenation recovery time indicate an impairment of oxygen utilization in subjects with FM. Although not tested in this study, these findings could reflect altered mitochondrial function. The pain and fatigue in subjects with FM have been found by others to be associated with mitochondrial dysfunction [6], as evidenced by decreased coenzyme Q_10 _(CoQ_10_), increased oxidative stress, as well as increased reactive oxygen species (ROS) production [6]. Mitochondrial dysfunction could also cause abnormal synthesis of adenosine-triphosphate (ATP), resulting in insufficient energy supply and muscle fatigue [6]. Accordingly, FM may impact mitochondrial oxidation to meet the increased metabolic demand during exercise, which would lead to a lower rOEF as observed in the present study.

On the other hand, following fatiguing exercise and cuff occlusion, extra oxygen is needed to compensate the oxygen loss in hemoglobin during exercise, and to oxidize lactate generated from anaerobic respiration [49]. This process is termed 'repaying the oxygen debt' [50]. Here, oxygen debt represents the oxygen deficit due to the imbalance between oxygen consumed by the tissue and that supplied via blood during exercise or muscle ischemia. Oxygenation recovery is dependent on the restoration of tissue microcirculation (that is, blood flow recovery) as well as the amount of oxygen debt to be repaid. We found in both exercise and occlusion protocols that the half-times of Δ[HbO_2_] and Δ[Hb] in subjects with FM were significantly longer than those in healthy controls (see Figure 5 and Figure 7), which is consistent with previous study findings using a cuff occlusion protocol or treadmill exercise [18,19]. The prolonged oxygenation recovery has been attributed to the imbalance between the oxygen supply and demand [18], although blood flow was not measured in those studies. In the present study, the FM group did not show a significant deficit in rBF recovery, so the prolonged oxygen recovery was independent of reactive hyperemia, but rather due to a higher oxygen debt. A higher oxygen debt in FM subjects was also observed in other studies [4,11,51,52], where subjects with FM were found to have a higher concentration of muscle lactate during anaerobic respiration. Fatiguing exercise [53] and muscle ischemic challenge [54] as used in the present study could induce extra muscle blood lactate in FM subjects. Lactate accumulation was recently proposed to be associated with muscle pain in FM subjects [55], although controversy remains [56]. Our findings suggest a future direction to explore the FM-induced alterations in mitochondrial function and lactate accumulation. An investigation of the relationships among oxygen kinetics, mitochondrial function and lactate dynamics are needed to further explore the origin of pain and fatigue in FM.

The present study is limited to the relative measurements of muscle blood flow, oxygen extraction fraction and oxygen consumption rate. However, some clinical outcomes, such as muscle capillary density, are found to be closely associated with the absolute values of muscle blood flow and oxygen consumption rate [57,58]. In addition, the blood flow index with a unit of cm^2^/s measured by DCS, needs to be calibrated to a classical blood flow unit of ml/min/100 ml for biological tissues. Another limitation is that we used the 6-second average data immediately after fatiguing exercise to represent the exercise-induced responses, as the optical data during exercise were contaminated by the muscle motion artifacts [26]. Although this method has been widely used in other exercise studies [46,47], it may generate measurement errors since muscle hemodynamics/metabolism change rapidly immediately after exercise. To overcome those limitations, we are currently designing calibration methods to obtain absolute tissue blood flow and VO_2_, as well as gating algorithms to reduce motion artifacts during exercise [59].

## Conclusions

We explored the application of a novel NIRS/DCS technique to simultaneously evaluate the responses of muscle blood flow, blood oxygenation and oxygen metabolism in subjects with FM and well-matched healthy controls during fatiguing exercise and muscle ischemic challenge. We found that FM resulted in less oxygen extraction in muscle during fatiguing exercise as well as longer oxygenation recovery following exercise and muscle ischemia. The results suggest an alteration of muscle oxygen utilization, which is possibly due to the altered mitochondrial function and/or lactate accumulation in the FM population. These findings verify our hypothesis that FM affects muscle hemodynamic/metabolic responses to fatiguing exercise and ischemic challenge, which can be noninvasively detected by the hybrid optical instrument. Notice that these conclusions are based on the data from relatively small samples (14 FMs and 23 healthy controls). A large subject pool would increase the statistical power of our measurement results. Combination of NIRS and DCS provides a unique tool to comprehensively evaluate tissue oxygen and flow kinetics in skeletal muscle.

## Abbreviations

BMI: body mass index; CAR: central activation ratio; CFS: chronic fatigue syndrome; DCS: diffuse correlation spectroscopy; Δ[Hb]: change in deoxyhemoglobin concentration; Δ[HbO_2_]: change in oxyhemoglobin concentration; ES: electrical stimulation; FM: fibromyalgia; [Hb]: deoxyhemoglobin concentration; [HbO_2_]: oxyhemoglobin concentration; HC: healthy control; IPAQ: international physical activity questionnaire; MVIC: maximal voluntary isometric contraction; NIRS: near-infrared diffuse optical spectroscopy; P-31 MRS: phosphorus magnetic resonance spectroscopy; PO_2_: partial pressure of oxygen; rBF: relative blood flow; rOEF: relative oxygen extraction fraction; rVO_2_: relative oxygen consumption rate; SE: standard error; StO_2_: tissue blood oxygen saturation; THC: total hemoglobin concentration; VAS: visual analog scale.

## Competing interests

The authors declare that they have no competing interests.

## Authors' contributions

YS carried out experiments, performed data analysis/interpretation and drafted the manuscript. KG participated in data analysis and drafted the manuscript. BS and DL carried out experiments and were involved in data interpretation. RS participated in data analysis. LC and CP conceived the study, participated in data interpretation and edited the manuscript. GY conceived the study, participated in data analysis/interpretation and drafted the manuscript. All authors read and approved the final manuscript.
